# Editorial: Diagnostic and predictive biomarkers in testicular germ cell tumors

**DOI:** 10.3389/fonc.2022.1027363

**Published:** 2022-09-13

**Authors:** João Lobo, Ricardo Leão

**Affiliations:** ^1^ Cancer Biology and Epigenetics Group, Research Center of IPO Porto (CI-IPOP)/RISE@CI-IPOP (Health Research Network), Portuguese Oncology Institute of Porto (IPO Porto)/Porto Comprehensive Cancer Centre (Porto.CCC), Porto, Portugal; ^2^ Department of Pathology, Portuguese Oncology Institute of Porto/Porto Comprehensive Cancer Centre (Porto.CCC), Porto, Portugal; ^3^ Department of Pathology and Molecular Immunology, School of Medicine & Biomedical Sciences, University of Porto (ICBAS-UP), Porto, Portugal; ^4^ Faculty of Medicine, University of Coimbra, Coimbra, Portugal

**Keywords:** biomarkers, testicular cancer (GCT), MicroRNAs, liquid biopsies, tumor microenvironment

Testicular germ cell tumors (TGCTs) comprise the most common solid malignancies afflicting young-adult men worldwide. Incidence is increasing due to the influence of environmental factors, which also contribute to subfertility issues, often co-occurring in these patients. We highlight three features of these tumors: they are developmental cancers, recapitulating several phenomena related to germ cell and embryonic development; they represent a model of “curable” cancer, with remarkable sensitivity to platinum-based chemotherapy; and they are very heterogeneous neoplasms, with several histological subtypes and particularities, requiring dedicated professionals and large cohorts for properly studying the whole spectrum of the disease (1).

There are several major challenges in the field of TGCTs: i) one relates to the limitations of currently available tools (classical serum tumor markers AFP, HCG and LDH, as well as imaging) for diagnosing and monitoring these patients. Classical markers are elevated in only 60% of TGCTs overall, being dependent on histological composition of the mass and being elevated in several other settings. These perform even more poorly for detecting disease relapses. Scrotal ultrasound confirms the presence of a testicular mass but does not reliably discriminate malignant TGCTs from other conditions causing testis enlargement (2). The definitive diagnosis is only made after surgical resection, on histopathological assessment, highlighting the need of better non-invasive biomarkers which capture this information upfront; ii) another challenge includes proper risk stratification of stage I patients, which comprise the vast majority of TGCTs at presentation. Current reports disclose substantial overtreatment of TGCT patients and related morbidity. Since these men are young individuals and will most likely survive their cancer, they will have to endure short- and long-term toxicity of platinum-based chemotherapy and radiation (3). This highlights the need for biomarkers that can indicate which patients should undergo active surveillance versus those that fully benefit from adjuvant systemic therapy, as well as biomarkers to detect minimal residual disease and, therefore, improve detection of relapse, which could lead to earlier salvage therapy; iii) in the setting of metastatic dissemination of disease, imaging of the retroperitoneum is also limited in accurately discriminating active post-chemotherapy germ cell malignancy, creating challenges in selecting the most appropriate treatment, again raising the need for non-invasive biomarkers that can improve assessment of these metastatic residual masses; iv) finally, patients developing cisplatin resistance often show poor prognosis and eventually die from the disease, as there are no approved therapies which are effective against this aggressive tumor phenotype. Studies show that cisplatin resistance is most likely multifactorial in nature, with contributions from several mechanisms and pathways (4), but this disease is challenging to investigate given its low prevalence. This raises the need of international cooperation for uncovering biomarkers predictive of resistance, so that this is anticipated, and also facilitating the design of alternative targeted therapies to offer these patients.

With this in mind, we have invited several recognized experts in the field of TGCTs to bring forth their contributions on these topics which are the main research questions left unanswered. We sincerely thank all researchers that have agreed to contribute to our Research Topic.

One section is dedicated to microRNAs as biomarkers. The microRNAs of the 371~373 cluster [in particular the miR-371a-3p (5)] are assuming themselves as the most promising non-invasive biomarkers of TGCTs, moving closer to clinical integration. This section counts with two contributions that further expand the applications of this biomarker. Dieckmann et al. explored the ability of miR-371a-3p quantification to discriminate viable disease after chemotherapy for metastatic seminoma patients, covering one of the aims of the Research Topic aimed at improving the assessment of post-chemotherapy residual masses. Also, showing that there is still room for some methodological improvements, Sequeira et al. propose a pipeline for quantifying miR-371a-3p using droplet digital PCR (ddPCR) in plasma samples, achieving a sensitivity of 94% and specificity of 100%. Additionally, they describe ddPCR optimization steps, comparing this pipeline with two classical real-time quantitative PCR technologies, and discussing the pros and cons of each methodology.

Another section of the Research Topic is devoted to the influence of the tumor microenvironment, more specifically infiltrating immune cells and inflammation on the prognosis of germ cell tumor patients. Evasion of immune destruction and tumor-promoting inflammation have indeed been added as hallmarks of cancer (6), and TGCTs are no exception. Bleve et al. provide a review on this topic, covering the impact of cancer-related inflammation and the PD-1/PD-L1 axis on patients’ outcome. Additionally, Kalavaska et al. performed immunophenotyping of peripheral blood leukocytes in chemo-naïve patients, looking for clinicopathological correlates and finding that specific immune cell subpopulations associated with poor survival.

A third section, dedicated to predictive biomarkers in the context of advanced disease treated with chemotherapy, is also included. Conduit et al. provide a meta-analysis of clinicopathological features that predict necrosis/fibrosis in retroperitoneal residual masses of non-seminoma patients, including histopathological features in the orchiectomy specimen (absence of teratoma, presence of seminoma), as well as serum tumor markers (normal HCG and AFP, elevated LDH) and size of the mass (>50% change in size, smaller residual mass size). Also, Rejlekova et al. identified features that predict development of choriocarcinoma syndrome (a severe complication, challenging to treat and with considerable mortality) in poor-risk germ cell tumor patients, including metastatic lung involvement >50% and ECOG PS ≥2.

Finally, this Research Topic also includes a review article by Burton et al., which addresses the important link between subfertility and TGCTs by looking into the role of Testis-expressed 101 (*TEX101*) and its interactome, covering the field of etiological factors for the emergence of TGCTs.

We hope that this Research Topic is of use for a wide audience, from basic scientists to medical professionals including urologists, medical oncologists, pathologists and radiologists, contributing to a better understanding of TGCTs. We are certain that an integrated approach, with international cooperation between professionals of different backgrounds, genome-wide methodologies and informative *in vitro* and *in vivo* models will continue to be key to improve the clinical care of TGCT patients.

## Author contributions

JL and RL drafted the manuscript and agreed on its final version.

## Conflict of interest

The authors declare that the research was conducted in the absence of any commercial or financial relationships that could be construed as a potential conflict of interest.

## Publisher’s note

All claims expressed in this article are solely those of the authors and do not necessarily represent those of their affiliated organizations, or those of the publisher, the editors and the reviewers. Any product that may be evaluated in this article, or claim that may be made by its manufacturer, is not guaranteed or endorsed by the publisher.
